# The first reported mortality from aluminum phosphide poisoning in Lebanon: a case report

**DOI:** 10.1186/s12245-024-00591-8

**Published:** 2024-02-06

**Authors:** Pierre Edde, Anthony El Kortbawi, Zeina Halabi, Nancy Sakr, Alondra Barakat, Tharwat El Zahran

**Affiliations:** 1 Notre Dame des Secours University Hospital Center, Street 93, Byblos, Postal Code 3, Lebanon / School of Medicine and Medical Sciences, Holy Spirit University of Kaslik, P.O.Box 446, Jounieh, Lebanon; 2 Department of Emergency Medicine, Faculty of Medicine, American University of Beirut Medical Center, Beirut, 1107 2020 Lebanon

**Keywords:** Aluminum phosphide, Insecticide, Intentional poisoning, Mortality, Emergency department

## Abstract

**Background:**

Aluminum phosphide is a commonly used pesticide, particularly in developing countries where uncontrolled insecticides and pesticides are commonly prevalent. Mortalities have been reported due to accidental and suicidal exposures to aluminum phosphide. To date, there has been no reported mortality case of aluminum phosphide in Lebanon. In addition, there is no specific antidote for aluminum phosphide toxicity and the treatment is mainly supportive. This is why awareness should be spread about this case to include it in the differential diagnoses and enhance prompt management and response in future encounters.

**Case presentation:**

A previously healthy 37-year-old male, presented to the emergency department of Notre Dame des Secours University Hospital Center for a suicidal attempt after ingesting 5 tablets of pesticide containing 56% aluminum phosphide an hour prior to presentation. Shortly after the presentation, the patient began deteriorating and became clinically unstable. The patient was then intubated and was started on sodium bicarbonate along with aggressive fluid resuscitation. The patient remained hypotensive even after giving vasopressors. He was then later admitted to the intensive care unit for further management. However, the patient further decompensated and developed multiorgan failure. This is the first case of mortality in Lebanon from aluminum phosphide toxicity.

**Conclusions:**

Emergency physicians should include aluminum phosphide toxicity in the differential diagnosis when dealing with patients ingesting unknown pesticides especially when they smell the characteristic garlic-like odor. The toxicity from ALP leads to multiorgan failure and death rapidly. Thus, it is of utmost importance to start early, and aggressive resuscitation given that there is no specific antidote.

## Background

Aluminum phosphide (ALP) is widely used as an insecticide and rodenticide in developed countries [1]. Suicide rates involving ALP are on the rise, particularly in developing nations like India, Sri Lanka, and Iran [2–4]. ALP is available in tablet form, often referred to as rice tablets, containing 56% aluminum phosphide. When it reacts with water or gastric fluids, it releases hydrogen phosphine gas (PH_3_) [5]. Phosphine gas has a garlic-like odor due to impurities [2, 3]. It inhibits cytochrome oxidase, leading to cellular hypoxia, asphyxia syndrome, multiorgan failure, shock, and death [2, 5]. There is no specific antidote for ALP poisoning, and management is primarily supportive. While several cases of successful management are reported in the literature [2, 6–10], severe toxicity can result in fatalities. This report describes the first ALP poisoning mortality case in Lebanon, along with a literature review.

## Case presentation

**Fig. 1:**
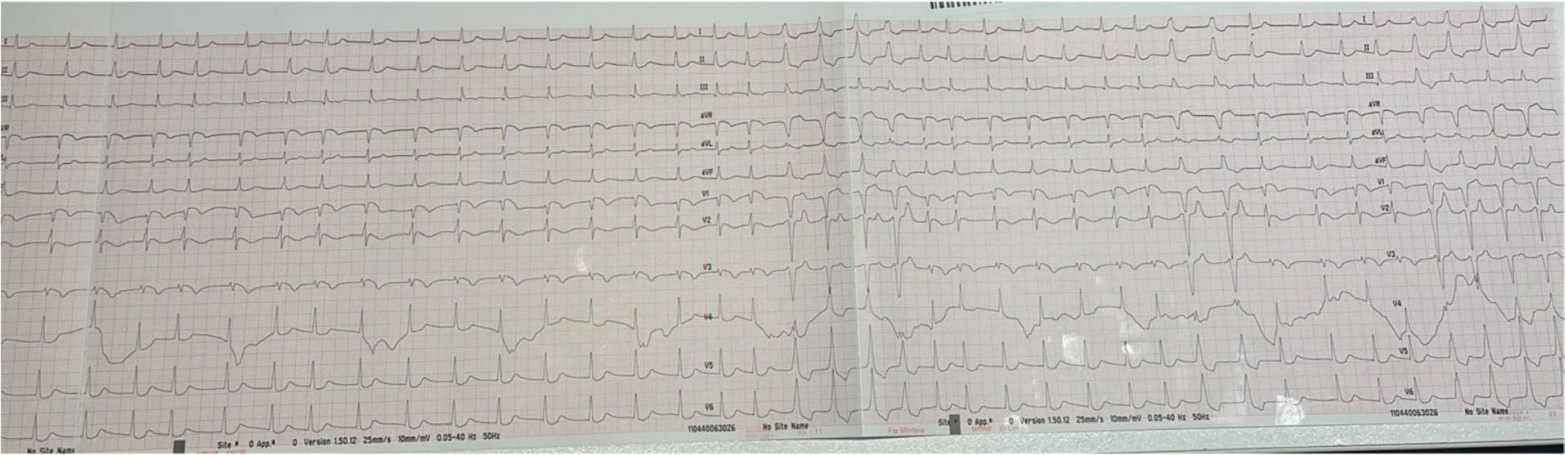
ECG of the patient

A previously healthy 37-year-old male arrived at the emergency department (ED) of Notre Dame des Secours University Hospital Center, through the emergency medical services (EMS), following a suicide attempt. He reported ingesting five pesticide tablets an hour before seeking medical attention. On arrival, he displayed anxiety, alertness, and abnormal vital signs, including a heart rate (HR) of 65 beats per minute, a blood pressure (BP) of 90/50 mmHg, a respiratory rate (RR) of 28 breaths per minute, pulse oximetry reading of 92% on room air, and a body temperature of 36.8 °C. He exhibited agitation and had a Glasgow Coma Scale (GCS) score of 10. Pupils were bilaterally mid-sized and reactive, and his skin was warm and dry. An electrocardiogram (ECG) indicated atrial fibrillation, ST depressions, and T wave inversions (Fig. 1). An intravenous fluid bolus was initiated and oxygen was administered. The toxicology service at AUBMC was consulted and activated charcoal (1 g/kg) was given due to recent ingestion of the pesticide.

Shortly after, the patient’s condition deteriorated, leading to hypotension (70/50 mmHg), desaturation (57%), and a GCS score of 4. Arterial blood gas (ABG) analysis on 5 L per minute of oxygen showed: pH 7.17, partial pressure of carbon dioxide (PCO2) 26.2 mmHg, partial pressure of oxygen (PO2) 143 mmHg, and bicarbonate (HCO3) 9.6 mmol/L. The patient was intubated to protect the airway, connected to a ventilator operating in A/C mode, with a tidal volume (TV) of 600 ml, respiratory rate (RR) of 28/min, a fraction of inspired oxygen (FiO2) set at 100%, and positive end-expiratory pressure (PEEP) of 5 cmH2O. Sodium bicarbonate was also administered. Subsequent ABG readings still indicated severe metabolic acidosis (pH 7.27, PCO_2_ 19.8 mmHg, HCO_3_ 9.2 mmol/L). Additional vials of sodium bicarbonate were administered, followed by a drip. Despite multiple normal saline boluses, the patient remained hypotensive, necessitating vasopressors. Repeat ABGs after 2 h also showed severe metabolic acidosis (pH 7.35, PCO_2_ 19.7 mmHg, HCO_3_ 11 mmol/L).

Initial blood tests showed high anion gap acidosis (AG 31, CO2 9 mmol/L), a creatinine level of 1.3 mg/dL, and sodium (Na) at 131 mmol/L (Table 1). A urine drug screen was not performed due to anuria since the presentation. Subsequent computed tomography (CT) scans of the brain, chest, abdomen, and pelvis revealed no visible intra or extra-axial cerebral hemorrhage, bilateral posterior-basal atelectasis with a small pleural effusion, multiple air bubbles in the anterior chest wall and mediastinum, infiltrates of mesenteric area, peripancreatic fat, and hepatic hilum, and thickened gallbladder wall containing dense content.

**Table 1:** Blood tests done during hospital stay

Blood tests	Day 1	Day 2	Day 3	References
WBCs	13.53	24.56	31.17	4.5–10.5 *1000/μL
Neutrophils	44	57	62	40–65%
Lymphocytes	46	37	36	25–40%
Hemoglobin	15.9	12	13.9	13–17 g/dL
Hematocrit	50	39	46	37–47%
Platelets	412,000	263,000	256,000	140–400*1000/μL
CRP	1	N/A	38	0–5 mg/L
Urea	26	29	N/A	17–46 mg/dL
Creatinine	1.3	2.1	3.9	0.8–1.2 mg/dL
Na	132	149	168	136–145 mmol/L
K	3.8	5.1	3.4	3.5–5.1 mmol/L
Cl	92	102	106	98–107 mmol/L
CO_2_	9	N/A	8	23–29 mmol/L
Ca	N/A	N/A	5.2	8.5–10.5 mg/L
Mg	N/A	N/A	8	1.6–2.5 mg/dL
P	N/A	N/A	1.9	2.4–4.8 mg/dL
CPK	N/A	352	1,351	24–204μ/L
AST	6	N/A	820	10–34μ/L
ALT	5	N/A	432	10–44μ/L
GGT	N/A	N/A	61	10–66μ/L
ALP	79	N/A	67	32–122μ/L
Direct bilirubin	0.4	N/A	1.9	0.2–1 mg/dL
Indirect bilirubin	0.8	N/A	1.5	0–0.2 mg/dL
Lipase	N/A	N/A	253	0–60 μ/L
Amylase	N/A	N/A	798	0–100 μ/L
Lactate	27.5	N/A	35.3	0.5–2.2 mmol/L

*CRP* C-reactive protein, *Na* sodium, *K* potassium, *Cl* chloride, *CO*_*2*_ carbon dioxide, *Ca* Calcium, *Mg* magnesium, *P* phosphorus, *CPK* creatine phosphokinase, *AST* aspartate aminotransferase, *ALT* alanine transaminase, *GGT* Gamma-glutamyl transferase, *ALP* alkaline phosphatase

The patient was then admitted to the intensive care unit (ICU), where aggressive fluid resuscitation was initiated, along with multiple vasopressors. Despite receiving over 15 L of 0.9% NaCl, the maximum dose of adrenaline, and more than 50 vials of HCO_3_, the patient remained hypotensive (systolic BP of 80 mmHg) and anuric, with continued severe metabolic acidosis (pH 7 and lactic acid level at 35 mmol/L). Cardiac ultrasound revealed a reduced left ventricular ejection fraction (LVEF) of 40% with paradoxical septal wall movement. After several hours, the patient developed significant bradycardia.

The following morning, the patient’s condition further deteriorated, resulting in multiorgan failure, including liver and renal failure (serum creatinine of 3.9 mg/dL, international normalized ratio (INR) of 3.4, aspartate aminotransferase (AST) of 820 μ/L, alanine transaminase (ALT) of 432 μ/L, bilirubin total/direct ratio of 1.9/1.5, ammonia level of 80 μmol/L, amylase of 798 u/L, and lipase of 253 μ/L). Despite withholding sedation, 100% FiO2, multiple vasopressors, and stress dose steroids, the patient remained comatose and hypotensive. Ultimately, he developed asystole and was pronounced dead 49 h after presentation.

**Fig. 2:**
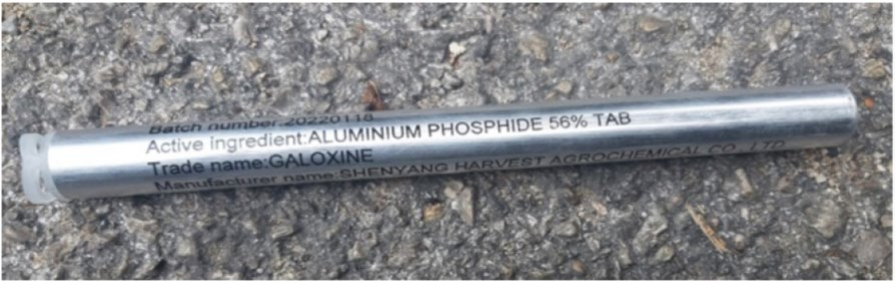
Picture of aluminum phosphide tablets

One day after the presentation, bystanders accompanying the patient in the ED confirmed that the ingested pesticide contained 56% of the toxic substance, narrowing it down to aluminum phosphide tablets (Fig. 2).

Gastric, urine, and blood samples were collected from the patient and sent to the environmental core laboratory at AUBMC for toxicological quantitative analysis. To analyze aluminum in samples, 2 mL of blood, urine, and gastric fluids were digested in an Anton-Paar Multiwave 5000 microwave digester with 9 mL nitric acid and 1 mL hydrogen peroxide. Quality control measures included blank spiked, dried oyster CRM, duplicate, and spiked samples. Post-digestion, aluminum content was analyzed using Agilent 7900 ICP-MS following EPA Method 200–8 (Determination of Metals and Trace Elements in Water and Wastes by ICP-MS-atomic emission spectrometry). Analysis results confirmed high levels of aluminum and phosphide in the serum and urine (Table 2). The serum aluminum level was significantly elevated compared to the normal levels (3440 μg/L; normal level is < 12 μg/L) [11].

**Table 2:** Aluminum level in samples

Sample type	Aluminum level (mcg/L)	Reference (mcg/L)
**Blood**	3440	12
**Urine**	7870	4–12

*LOQ* 0.015 μg/L, *UR* R ± 19% of R

## Discussion

The global burden of pesticide poisoning is rising, particularly in developed nations [12]. It has been declared the most common suicide method by the World Health Organization (WHO) due to its high case fatality rate, which can exceed 70% [4].

In Lebanon, a few cases of metal phosphide poisoning have been reported, such as zinc phosphide poisoning, which led to gastrointestinal disturbances and ICU admission, but the patient survived [13]. This is the first reported ALP poisoning case in Lebanon, resulting in multiorgan failure and death. ALP poisoning is known to be more severe, often leading to mortality [2, 14].

The lethal dose of ALP is 20 mg/kg, or approximately 150 to 500 mg, considering tablet exposure to humidity [2, 15, 16]. In this case, the patient’s weight was 110 kg, and he ingested approximately 15 g (around 73 mg/kg) of ALP, a lethal toxic dose. Patients typically succumb within 24 h post-exposure [1], but in this case, the patient died after 49 h.

Diagnosis usually relies on clinical suspicion and a history of ingestion [17]. Testing for phosphine in the blood is impractical due to its rapid oxidation into phosphite [18]. Qualitative color tests, such as the silver nitrate test, are used to detect phosphine in biological samples. In this patient, quantitative tests were performed to measure aluminum levels in gastric fluid, blood, and urine samples (Table 2).

The gastrointestinal tract (GI) is the first affected organ with oral ALP ingestion [15]. Although the patient initially presented with no GI symptoms, he later developed liver failure with elevated transaminases, INR, and pancreatic enzymes. Liver failure has been reported in ALP poisoning, with autopsy findings showing fatal cytoplasmic vacuolization of liver cells [17].

**Fig. 3:**
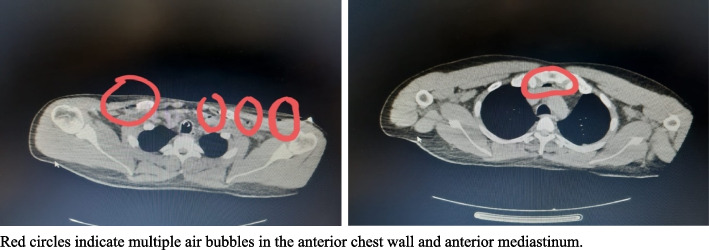
CT chest of the patient

Involvement of cardiac and pulmonary systems leads to circulatory collapse, brain anoxia, GCS deterioration, and eventual death [16, 17]. The patient experienced hypoxic respiratory failure, requiring intubation, high FiO2, and PEEP. His CT chest revealed pleural effusions a few hours after presentation (Fig. 3). Adult respiratory distress syndrome has been reported with ALP overdose [1, 17]. The patient’s cardiac function declined, with an ejection fraction of 40% and paradoxical septal wall movement. ECG findings showed atrial fibrillation, ST depressions, and T wave inversions, potentially linked to myocardial cell necrosis, which can cause conduction defects, wall hypokinesia, tachy- and brady-dysrhythmias [1, 16].

Severe metabolic and lactic acidosis are also common in ALP poisoning [1, 17]. The patient experienced both metabolic and lactic acidosis with tissue hypoperfusion. ALP poisoning can lead to electrolyte imbalances like hypokalemia, hypo- or hyper-magnesemia, and hypoglycemia [1]. Acute renal failure with oliguria, as observed in our patient, is also reported [1].

No specific antidote for ALP poisoning exists, and treatment is primarily supportive. However, some cases have shown favorable outcomes with various approaches. Activated charcoal may be considered for oral ALP ingestion if a patient presents within an hour with no contraindications. Nonetheless, activated charcoal and potassium permanganate may have limited benefits due to the lack of molecular interaction in metal phosphide poisoning [13]. Antioxidants, like N-acetyl cysteine (NAC), are proposed for ALP poisoning treatment.

Mortality from ALP poisoning is high, reaching 60% [19]. Variables predicting mortality in our patient included elevated serum creatinine, severe metabolic acidosis, low serum bicarbonate, the need for mechanical ventilation, and vasopressor administration [19].

In conclusion, this is the first reported ALP poisoning mortality in Lebanon. Emergency physicians should be vigilant for aluminum phosphide poisoning in patients with intentional overdose, rapid deterioration, multiorgan failure, and shock. Physicians should be prepared for rapid patient decompensation and be ready to provide aggressive resuscitation and a higher level of care.

## Data Availability

The datasets used and/or analyzed during the current study are available from the corresponding author on reasonable request.
